# Third COVID-19 vaccine dose for people with multiple sclerosis who did not seroconvert following two doses of BBIBP-CorV (Sinopharm) inactivated vaccine: A pilot study on safety and immunogenicity

**DOI:** 10.3389/fimmu.2023.952911

**Published:** 2023-02-21

**Authors:** Nahad Sedaghat, Masoud Etemadifar, Noushin Lotfi, Farnaz Sayahi, Ahmad Chitsaz, Mehri Salari, Alireza Ghasemi Movaghar

**Affiliations:** ^1^ Alzahra Research Institute, Alzahra University Hospital, Isfahan University of Medical Sciences, Isfahan, Iran; ^2^ Network of Immunity in Infection, Malignancy, and Autoimmunity (NIIMA), Universal Scientific Education and Research Network (USERN), Isfahan, Iran; ^3^ Department of Neurosurgery, School of Medicine, Isfahan University of Medical Sciences, Isfahan, Iran; ^4^ Department of Immunology, School of Medicine, Isfahan University of Medical Sciences, Isfahan, Iran; ^5^ Isfahan Research Committee of Multiple Sclerosis (IRCOMS), Isfahan Multiple Sclerosis Center, Isfahan, Iran; ^6^ Department of Neurology, School of Medicine, Isfahan University of Medical Sciences, Isfahan, Iran; ^7^ Functional Neurosurgery Research Center, Shohada Tajrish Comprehensive Neurosurgical Center of Excellence, Shahid Beheshti University of Medical Sciences, Tehran, Iran

**Keywords:** COVID-19, multiple sclerosis, vaccine immunogenicity, disease-modifying therapies (DMTs), BBIBP-CorV

## Abstract

**Background:**

People with multiple sclerosis (pwMS) on anti-CD20 therapies (aCD20) and fingolimod have shown inadequate humoral responses to COVID-19 vaccines.

**Objective:**

The objective of the study was to pilot larger studies by demonstrating the safety and comparing the immunogenicity of different types of third doses in seronegative pwMS after two doses of BBIBP-CorV inactivated vaccine.

**Methods:**

In December 2021, subject to receiving their third dose, being COVID-19-naiive, and receiving no corticosteroid within two months, we measured the level of anti-SARS-CoV-2-Spike IgG in pwMS seronegative after two shots of BBIBP-CorV inactivated vaccine.

**Results:**

We included 20/29 pwMS who received adenoviral vector (AV), 7/29 who received inactivated, and 2/29 who received conjugated third doses. No serious adverse events were reported two weeks post-third dose. The pwMS receiving AV third doses showed significantly increased IgG concentrations, while only the ones *not* on aCD20 and fingolimod responded to inactivated third doses. An ordinal logistic multivariable generalized linear model indicated that age (per year β: −0.10, P = 0.04), type of disease-modifying therapy (aCD20 β: −8.36, P <0.01; fingolimod β: −8.63, P = 0.01; others: reference), and type of third dose (AV or conjugated β: 2.36, P = 0.02; inactivated: reference) are predictive of third dose immunogenicity among pwMS who remain seronegative after two shots of BBIBP-CorV vaccine. Statistical significance was not achieved for variables sex, MS duration, EDSS, duration of DMT, duration of third dose to IgG test, and duration from last aCD20 infusion to third dose.

**Conclusion:**

This preliminary pilot study highlights the need for further research to determine the optimal COVID-19 third dose vaccination strategy for pwMS living in areas where BBIBP-CorV vaccine has been used.

## 1 Introduction

Among people with multiple sclerosis (pwMS) on sphingosine-1-phosphate receptor modulators (S1PRM) and anti-CD20 therapies (aCD20) primed with mRNA COVID-19 vaccines, evidence shows persistent vaccination failure after mRNA third doses (1), while data on third-dose immunogenicity remain scarce for those receiving other types of third doses—or any other primary series than adenoviral vector (AV) or mRNA. Given the vast global usage of inactivated vaccines as primary series, particularly in densely-populated developing areas, the determination of third dose safety and immunogenicity among immunocompromised people who fail to respond to the inactivated primary series is of relevance for future evidence-driven policy making and practice. Hence, in order to facilitate more research on the subject, we decided to reidentify from our previous study (2) the pwMS who remained seronegative after two doses of BBIBP-CorV, determine the frequency of serious adverse events, measure the anti-SARS-CoV-2 IgG levels among the ones who received their third dose, and investigate the effect of different variables on third-dose immunogenicity among them.

## 2 Methods and results

### 2.1 Design, settings, and participants

As an extension of an observational retrospective cohort study conducted in December 2021 in Isfahan, Iran, we identified 49 adults with definitive MS who received two doses of BBIBP-CorV but remained seronegative from our previous study (2) and contacted them, asking if they had received any kind of third dose of a COVID-19 vaccine. Among them, 21 women and eight men (mean age [SD]: 40 years [10.60]) were COVID-19-naiive—defined as having no history of: i) clinical illnesses compatible with COVID-19, ii) contact with suspected or confirmed COVID-19 patients, or iii) COVID-19 diagnosis based on the available laboratory and imaging methods—and did not receive corticosteroids within two months of their third dose. 12 were receiving aCD20, eight fingolimod, four teriflunomide, two glatiramer acetate (GA), and one dimethyl fumarate (DMF), without any change in DMT regimen in the past year. Their further demographics and MS-related characteristics are interpretable from **Table 1**.

**Table 1:** Characteristics of participants.

Variable	Heterologous^1^ third dose (n = 22)	Homologous^2^ third dose (n = 7)	P-value
Mean Age (Years) [SD]	39.04 (11.28)	43 (8.08)	0.40^#^
Sex (n, %) [M:F]	7 (31.82): 15 (68.18)	1 (14.29): 6 (85.71)	0.36^@^
Mean MS duration (Years) [SD]	8.73 (5.98)	14.29 (4.57)	0.03^#^
Median EDSS (Range)	2.25 (1–4)	2 (1–3.5)	0.81*
DMT (n, %)			0.56^@^
* aCD20	8 (36.36)	4 (57.14)	
* Fingo	7 (31.82)	1 (14.29)	
* Other	7 (31.82)	2 (28.57)	
Median duration on current DMT (years) [Range]	5 (1–10)	5 (4–7)	0.27*
Median (Range) of weeks from:			
*2nd dose to 3rd dose	23.5 (16–28)	23 (11–25)	0.28*
* 2nd dose to subsequent IgG test	7 (2–18)	5 (3–9)	0.47*
* 3rd dose to subsequent IgG test	3 (2–6)	3 (2–6)	0.55*
* aCD20 infusion to 3rd dose (n = 12)	14.5 (5–22)	11 (9–12)	0.26*
Median anti-SARS-CoV-2-Spike IgG (Range)	31.75 (0.14– >100)	1.75 (0.17–47.10)	0.048*

^#^Student’s T-test; ^@^Pearson Chi^2^; *Mann–Whitney U test. Assumptions of normality of distributions were tested using the Kolmogorov–Smirnov method; variables, the distribution of values of which passed the Kolmogorov–Smirnov test, are reported with mean and SD, and compared using parametric statistics; others are reported with median and range, and compared using non-parametric statistics.

1. AstraZeneca ChAdOx1 nCoV-19 (n = 20) and Pasteur Institute of Iran PastoCovac (n = 2).

2. Sinopharm BBIBP-CorV.

SD, standard deviation; M, male; F, female; MS, multiple sclerosis; EDSS, expanded disability status scale; DMT, disease-modifying therapy; aCD20, anti-CD20 therapies; Fingo, fingolimod.

Furthermore, information was collected regarding the date and type of their third dose and the development of any serious adverse events following their third dose, as defined by the US Food and Drug Administration (FDA) (3)^1^. A total of 20/29 of the participants received AV (ChAdOx1 nCoV-19, AstraZeneca), 7/29 inactivated (BBIBP-CorV, Sinopharm), and 2/29 conjugated (PastoCovac, Pasteur Institute of Iran) third doses. The participants receiving inactivated third doses had higher disease durations than the ones receiving AV/conjugated third doses (mean diff. 5.56 years, P = 0.03); their other known features were similar (**Table 1**).

### 2.2 Post-third dose safety and antibody responses

None of the participants reported any serious adverse events—including MS relapses or pseudorelapses—from the administration of their third dose until two weeks later. At least two weeks after their documented third dose, blood samples were obtained from the participants and used for quantification of post-third dose anti-SARS-CoV-2-Spike IgG—which corresponds to neutralizing activity against variants of concern (VOC) (4–6) with an ELISA kit (Pishtazteb Diagnostics, Iran), in accordance with manufacturer instructions (7) and with methods previously described (2). The IgG levels above the kit’s upper limit of quantification (ULoQ) were regarded as >100 RU/ml without precise quantification due to restrictions in rerunning the assays with serial dilutions of samples.

#### 2.2.1 Antibody responses in pwMS on aCD20

**Figure 1:**
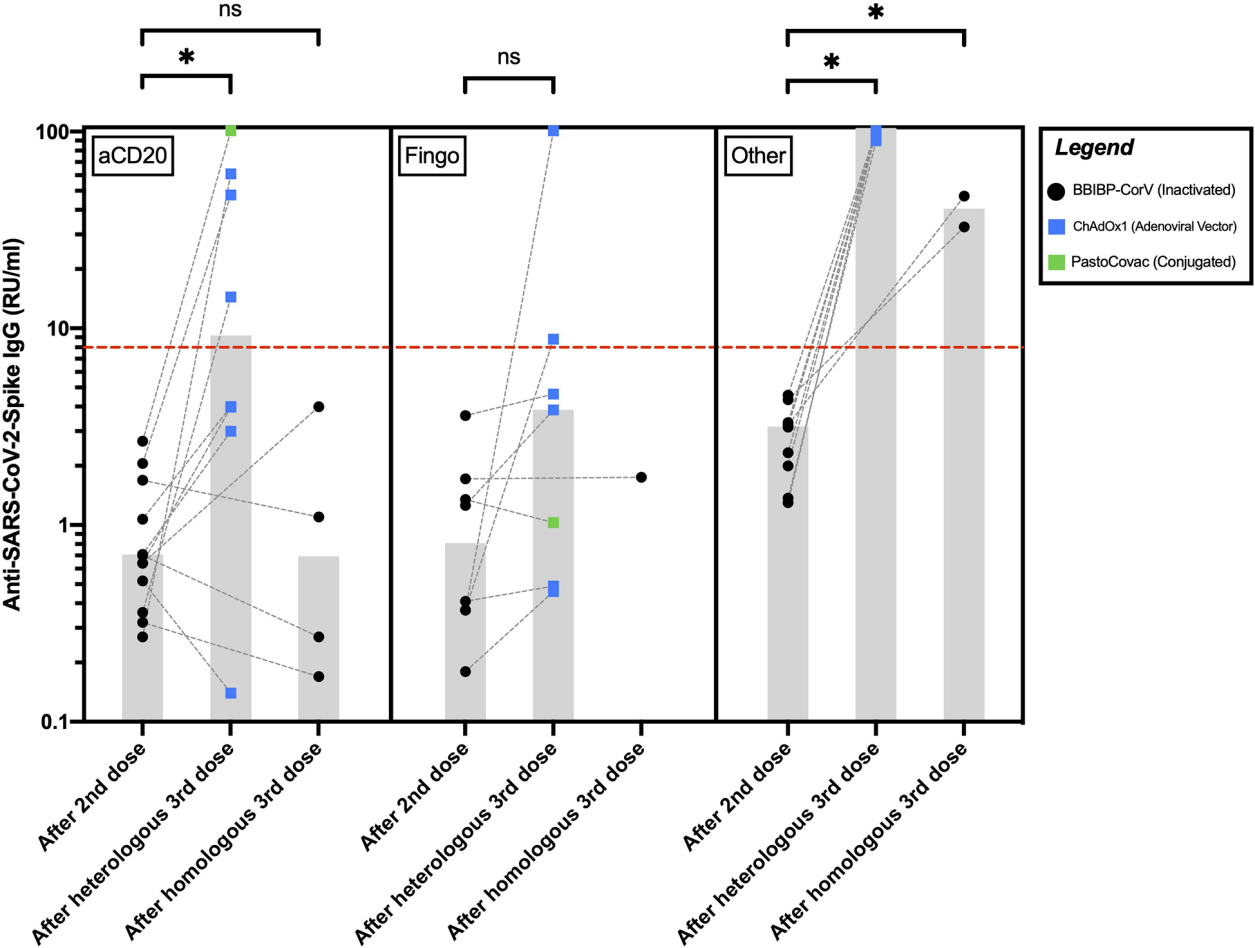
Anti-SARS-CoV-2-Spike IgG levels in pwMS receiving anti-CD20 therapies, fingolimod, or other disease-modifying therapies after their second and third COVID-19 vaccine doses. The red hyphenated line corresponds to the seropositivity cut-off index. The gray bars correspond to the median of IgG levels. *P <0.05; ns, P >0.05. aCD20, anti-CD20 therapies; Fingo, fingolimod; RU/ml, relative units per milliliter.

Among the pwMS on aCD20, 4/8 (50%) of the ones receiving AV/conjugated third doses and none of the four who received inactivated third doses seroconverted after their third dose. Anti-SARS-CoV-2-Spike IgG levels increased significantly in the aCD20-treated participants after receiving AV/conjugated third doses (P=0.01^2^) but did not differ significantly in the ones receiving inactivated third doses (P = 0.87^a^) (**Figure 1**).

#### 2.2.2 Antibody responses in pwMS on fingolimod

Among the pwMS on fingolimod, 2/7 (28%) of those receiving AV/conjugated third doses seroconverted after their third dose. The only fingolimod-receiving participant who received an inactivated third dose did not seroconvert. Anti-SARS-CoV-2-Spike IgG levels increased insignificantly after AV/conjugated third doses among the fingolimod-receiving pwMS (P = 0.07^a^). Statistical significance was reached after excluding the single fingolimod-treated participant who received a conjugated third dose (P = 0.03^a^).

#### 2.2.3 Antibody responses in pwMS on other DMTs

All the participants on other DMTs seroconverted following their third dose. Among them, compared to the ones receiving inactivated third doses, anti-SARS-CoV-2-Spike IgG levels were significantly higher in those receiving AV third doses (P = 0.03^b^) (**Figure 1**).

#### 2.2.4 Further analysis

A multivariable generalized linear model controlling for the confounding effect of variables age, sex, MS duration, expanded disability status scale (EDSS), DMT, and the interaction term of DMT and duration of being on the DMT indicated a significant (P <0.05) effect of age, DMT type, and third dose type on the post-dose 3 increase in IgG levels (**Table 2**); the possible effects of other mentioned variables were not statistically confirmed (P >0.05).

**Table 2:** Multivariable generalized linear model.

Variable (reference)	Multivariable GLM (n=29, ordinal logistic response, outcome: rank of increase in anti-SARS-CoV-2-Spike IgG [RU/ml] after third dose)	
	B (SE)	P-value
**Age (per year)**	-0.10 (0.05)	0.04
**Female sex (male)**	0.19 (0.92)	0.84
**MS duration (per year)**	-0.13 (0.11)	0.25
**EDSS (per score)**	0.62 (0.54)	0.25
**DMT type (Other)**		
** –aCD20**	-8.36 (3.01)	<0.01
– **Fingo**	-8.63 (3.48)	0.01
**Duration of receiving DMT (per year)**		
– **aCD20**	0.74 (0.43)	0.09
– **Fingo**	0.57 (0.53)	0.28
– **Other**	0.18 (0.31)	0.57
**Heterologous third dose (homologous)**	2.36 (1.02)	0.02
**Duration from third dose to phlebotomy (per week)**	-0.99 (0.54)	0.07

GLM, generalized linear model; B, beta coefficient; SE, standard error; MS, multiple sclerosis; EDSS, expanded disability status scale; DMT, disease-modifying therapy; aCD20, anti-CD20 therapies; Fingo, fingolimod.

## 3 Conclusion

Our study demonstrates that the immunogenicity of the third COVID-19 vaccine dose could be safely studied in adult pwMS who received two doses of an inactivated vaccine but remained seronegative. In line with studies among non-MS adults, which have consistently demonstrated the superiority of AV or mRNA third doses over inactivated ones in terms of humoral immunogenicity and clinical effectiveness against the VOC (8–12), our results hint that AV third COVID-19 vaccine doses may be of more benefit than inactivated ones for pwMS who received two doses of an inactivated vaccine but remained seronegative. Third dose studies are currently scarce among pwMS who received inactivated primary series; however, such studies among pwMS receiving mRNA or AV primary series have suggested that less time from the last aCD20 infusion and being on S1PRM and aCD20 DMTs blunt seroconversion rates (1, 13–15). The current study lacked adequate statistical power to support the former but could be considered supportive of the latter statement. Nevertheless, administration of third booster doses, although not as much as other pwMS, could be considered beneficial for pwMS on S1PRM and aCD20 (14) but its cost-effectiveness in the current state of the pandemic remains to be investigated.

## 4 Limitations

Although some of our findings are unlikely to be explained merely by chance as interpreted from hypothesis testing and statistical significance, our study was conducted on a limited number of participants; therefore, the certainty of our findings is very low. Also, due to our small sample size, the effect of the aCD20 infusion-to-third dose period could not be investigated. The results of the current study have implications for future larger studies on the subject; conclusions from our study that might affect real-world practices are subject to validation by studies with larger sample sizes. We encourage future researchers to account for the limitations of this study by recruiting a larger sample size, collecting data on mild to moderate adverse events, quantifying measures above ULoQ, using T cell and/or neutralization assays, and including previously-seropositive participants who might have been subject to immunity waning—especially the ones on aCD20, cladribine, and alemtuzumab.

## Data availability statement

The datasets presented in this article are not readily available because of the privacy protection of the participants. The generated data in this study will be available to qualified investigators upon a reasonable request from the corresponding author and in context of a non-disclosure agreement. Requests to access the datasets should be directed to nahad.sedaghat@gmail.com.

## Ethics statement

The studies involving human participants were reviewed and approved by Research Ethics Committee of School of Medicine, Isfahan University of Medical Sciences. The patients/participants provided their written informed consent to participate in this study.

## Author contributions

NS: writing - initial draft; formal analysis; conceptualization. ME: conceptualization; resources; supervision. NL: resources; supervision. FS: data curation, resources. AC: resources. MS: writing - review and editing. AG: data curation. All authors contributed to the article and approved the submitted version.

## Footnotes

1 No data was collected on development of mild to moderate adverse events.

2 Wilcoxon matched-pairs signed rank test.
